# Systemic sarcoidosis with bone marrow involvement responding to therapy with adalimumab: a case report

**DOI:** 10.4076/1752-1947-3-8573

**Published:** 2009-07-29

**Authors:** Supen R Patel

**Affiliations:** 1 Carolina Health Care, East Cheves Street, Florence, SC 29503, USA

## Abstract

**Introduction:**

Sarcoidosis is an inflammatory disorder characterized by the presence of non-caseating granulomas in affected organs. The presence of CD4-positive T lymphocytes and macrophages in affected organs suggests an ongoing immune response. Systemic corticosteroids remain the mainstay of treatment, but therapy is often limited by adverse effects. This is the first report of the use of adalimumab (HUMIRA^®^, Abbott Laboratories, North Chicago, IL, USA), an anti-tumor necrosis factor monoclonal antibody, in a patient with systemic sarcoidosis with bone marrow involvement.

**Case presentation:**

A 42-year-old African-American man with a medical history significant for hypertension and diabetes mellitus presented with anemia and thrombocytopenia of two months duration. The patient underwent physical examination, bone marrow aspiration and biopsy, chest X-ray, acid-fast bacilli stain, computed tomography with contrast, and additional laboratory tests. He was diagnosed with systemic sarcoidosis with splenomegaly and bone marrow involvement. Drug therapy included prednisone, which had to be discontinued owing to adverse effects, and adalimumab.

**Conclusion:**

This is the first report describing the use of adalimumab in a patient with systemic sarcoidosis with bone marrow involvement. Tumor necrosis factor antagonism with adalimumab was efficacious and well-tolerated in this patient and may be considered as a treatment option for similar cases.

## Introduction

Sarcoidosis is an inflammatory disorder characterized by the presence of non-caseating granulomas in affected organs [1]. The etiology is unclear, but the presence of CD4-positive T lymphocytes and macrophages in affected organs suggests an ongoing immune response [1]. The clinical course of sarcoidosis is highly variable. One organ may be involved or systemic disease may be present. Bone marrow involvement is rare, and isolated extrapulmonary sarcoidosis occurs in less than 5% of cases [2,3]. The disease can be self limiting or chronic [1]. There is no universally accepted treatment for sarcoidosis. Systemic corticosteroids remain the mainstay of treatment, but therapy is often limited by adverse effects [1,4,5]. This article describes the first report of the use of adalimumab (HUMIRA®, Abbott Laboratories, North Chicago, IL, USA), an anti-tumor necrosis factor (anti-TNF) monoclonal antibody, in a patient with systemic sarcoidosis with bone marrow involvement.

## Case presentation

A 42-year-old African-American man was referred to our offices with a 60-day history of decreased hemoglobin (5.52 mmol/L) and an accompanying platelet count of 132 × 10^9^/L. A follow-up complete blood count in our office revealed a further decrease in hemoglobin (4.41 mmol/L) in addition to a low white blood cell count (2.9 × 10^9^/L), with a platelet count of 163 × 10^9^/L and mean cell volume of 77.3fL (Table 1). The patient reported weight loss of eight pounds in the past two and a half months, owing to lack of appetite. He reported no fever, night sweats, hematochezia, melena, epistaxis, or other systemic problems. Despite obvious anemia, the patient had no weakness or shortness of breath. His medical history was significant for hypertension, a two year history of diabetes mellitus, and a sickle-cell trait; he was otherwise in good health. There was no significant family or social history. A physical examination was notable for a firm spleen about 4 cm below the costal margin. The patient's stools were hemoccult negative. No petechiae, cyanosis, clubbing, neurologic focality, skin rash, or joint effusions were noted.

**Table 1:** Laboratory parameters

Point in time		Laboratory parameter							
		Therapy	Hb (mmol/L)	WBC (×10^9^/L)	PLT (×10^9^/L)	MCV (fL)	Glu (mmol/L)	CRP (mg/L)	Sed (mm/hour)
2 months before referral		None	5.52		132				
Referral		None	4.41	2.9	163	77.3			
Diagnosis		None^a^	4.03	3.7	191	77.4			
Post-diagnosis (wk)	1	Pred	4.34	3.9	220	79.7			
	2	Pred	4.41	4.0	201	80.0	14.6	27.0	67
	9	ADA^b^	5.96	2.7	142	79.5	14.3	25.0	41
	20	ADA^c^	5.46	3.3	179	78.0	5.8	33.0	105
	24	None^d^	4.59	3.0	147	79.7	4.9	36.0	80
	31	None^d^	4.41	3.8	174	80.9		34.0	92
	36	ADA^e^	4.10	3.7	152	84.3	5.9	30.0	50
	40	ADA	4.47	3.4	170	82.6	4.2	23.0	66
	45	ADA	5.03	3.6	177	83.4		9.0	63
	49	ADA	6.02	4.2	165	80.9	4.3	6.0	63
	53	ADA	5.96	3.7	146	77.2		4.0	14
	57	ADA	6.4	4.0	122	77.5	117	4.0	47

^a^Began prednisone at 20 mg/day;

^b^second dose of adalimumab, tapered off prednisone;

^c^7 doses of adalimumab, completely off prednisone;

^d^patient discontinued adalimumab for 9 weeks; adalimumab was restarted at post-diagnosis week 33;

^e^three doses of second course of adalimumab.

Abbreviations: ADA, adalimumab; CRP, C-reactive protein; Glu, glucose; Hb, hemoglobin; MCV, mean cell volume; PLT, platelets; Pred, prednisone; Sed, sedimentation rate; WBC, white blood cells.

From the laboratory work, there was no evidence of hemolysis to account for the anemia with splenomegaly. The differential diagnosis was broad and included malignancies, such as hairy cell leukemia, splenic villus lymphoma, and myelodysplasia with myelofibrosis. A bone marrow aspirate and biopsy were ordered. Special stains for acid-fast bacillus and fungi were negative. Flow cytometry revealed no evidence of acute leukemia, lymphoma, plasma cell dyscrasia, or immunophenotypic changes associated with myelodysplasia. Blast cells, monocytes, myeloid cells, and lymphocytes were not relatively increased and had normal antigen expression. Results showed normocellular bone marrow with non-caseating granulomas, with no immunophenotypic abnormalities, thus leading to a diagnosis of systemic sarcoidosis with splenomegaly and bone marrow involvement.

During the office visit on the day the diagnosis was discussed with the patient, a chest X-ray was performed and showed no evidence of lung nodules or hilar adenopathy. Angiotensin-converting enzyme concentrations were checked and were within the normal range. The patient reported slight weakness but no shortness of breath, exertional dyspnea, or chest pain. However, the hemoglobin concentration had decreased to 4.03 mmol/L. Steroid therapy was initiated with a prednisone dosage of 20 mg/day. Two weeks later, results from hematology tests revealed a slight improvement in anemia; however, blood glucose concentrations were elevated (14.6 mmol/L; Table 1). The patient had gained a small amount of weight but reported fatigue and night sweats. Computed tomography (CT) with contrast was performed to rule out liver involvement; results showed moderate splenomegaly (the markedly enlarged heterogeneous spleen measured 20 cm in length × 20 cm obliquely × 10 cm obliquely), with no noted hepatomegaly.

Four weeks post-diagnosis, the patient reported polyarthritis symptoms and discomfort. Sedimentation rate and C-reactive protein concentrations were elevated and the patient continued to be anemic and leukopenic (Table 1). Glucose concentrations were elevated; thus, prednisone therapy was decreased to a dosage of 15 mg/day. Because of extensive spleen involvement and his history of diabetes, and because therapy with TNF antagonists has been shown to be efficacious in the treatment of sarcoidosis [4]-[12], treatment with adalimumab 40 mg every other week was prescribed. Before initiation of adalimumab therapy, a purified protein derivative (PPD) skin test was performed and results were negative for tuberculosis.

Two weeks after adalimumab was initiated, the patient's anemia was markedly improved, as evidenced by an elevation in hemoglobin concentration to 5.96 mmol/L (Table 1). Because of elevated blood glucose concentrations and a clinical response to adalimumab therapy, prednisone tapering was initiated. Over the next three and a half months of adalimumab therapy, the hemoglobin concentration gradually decreased but remained above baseline (Table 1). A physical examination performed 5 months post-diagnosis revealed that the patient's spleen was palpable approximately 2 cm below the costal margin.

**Figure 1:**
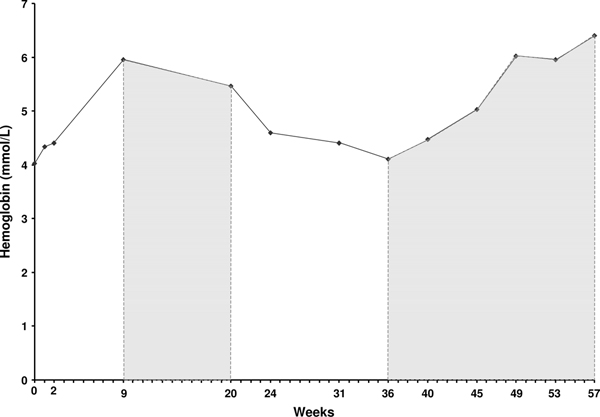
**Concentrations of hemoglobin, white blood cells, and platelets over time**. Concentrations of hemoglobin (mmol/L), white blood cells (×10^9^/L), and platelets (×10^11^/L) at baseline and during the course of the patient's treatment. At diagnosis (Week 0), the patient began a prednisone regimen that was tapered after initiation of adalimumab treatment. Shaded areas indicate the first and second courses of adalimumab treatment. The patient discontinued adalimumab treatment between the 24th and 32nd weeks; adalimumab treatment was reinitiated at Week 33. Hbg, hemoglobin; PLT, platelets; WBC, white blood cells.

Approximately 8 months post-diagnosis, it was discovered that the patient had discontinued adalimumab 9 weeks previously; the patient said he thought he was supposed to stop the medication. His hemoglobin concentration had decreased to 4.41 mmol/L. Adalimumab was reinitiated. The hemoglobin concentration continued to decrease over the next 5 weeks (Table 1), but after approximately five doses of the second course of adalimumab, his hemoglobin concentration increased to 4.47 mmol/L and his anemia continued to improve over the next several months. By the 49th week post-diagnosis, the patient's spleen was no longer palpable and a chest X-ray was negative. His initial weight loss was completely resolved. After approximately 18 doses of the second course of adalimumab therapy, his hemoglobin concentration had increased to 6.40 mmol/L, the highest concentration since therapy began (Table 1 and Figure 1). Follow-up CT scans revealed marked decreases in spleen size post-diagnosis; spleen size was 13 × 6.4 cm 18 months after the first CT scan and 11.6 × 5.6 cm 25 months after the first CT scan.

## Discussion

Although systemic corticosteroids are generally the primary treatment of sarcoidosis, there was concern about glycemic control with corticosteroid use in this patient because of his diabetes; nonetheless, a trial with prednisone was considered to be worthwhile. When blood glucose concentrations became elevated, however, other treatment options were considered. Methotrexate is the most widely studied steroid-sparing treatment for sarcoidosis and has been reported as useful for various organ systems affected by sarcoidosis [13]. However, methotrexate was not considered in this patient for numerous reasons: 1) methotrexate can have cytotoxic effects on bone marrow [14], 2) methotrexate is contraindicated in patients with significant anemia [14], and 3) there is an increased risk of hepatotoxicity when methotrexate is used in patients with diabetes [13]. Similarly, other medications with hematologic safety concerns (for example, anemia, leukopenia, neutropenia associated with leflunomide and mycophenolate mofetil) were not considered for administration in this patient [15,16]. Serious adverse events associated with anti-TNF therapy include infection and malignancy; however, based on 10 years of clinical trial experience with adalimumab in more than 19,000 patients across six different immune-mediated inflammatory diseases, cumulative rates of serious infections have remained stable over time, and malignancy rates are similar to those in the general population [17].

There is evidence that TNF is involved in the pathogenesis of sarcoidosis, and there have been numerous reports of the successful treatment of sarcoidosis with TNF antagonists [4]-[12]. Early experience with TNF antagonists in sarcoidosis has been primarily based on the use of infliximab for cutaneous and extracutaneous sarcoidosis [4,6,8,9,11]. Etanercept has not been as effective; a small Phase II trial for the treatment of pulmonary sarcoidosis was terminated early because of a large number of treatment failures [18]. Adalimumab is a subcutaneously administered, fully human immunoglobulin G_1_ monoclonal anti-TNF antibody. In case reports, adalimumab has been used successfully in patients with treatment-resistant sarcoidosis [10,12]. TNF-antagonist treatment appears to be particularly useful when steroid treatment has failed [4,6,9]. In this patient, adalimumab was efficacious; hemoglobin concentrations increased after initiation of adalimumab (Figure 1) and other blood values began to normalize. It is difficult to determine the precise extent to which hemoglobin concentrations changed because the patient discontinued adalimumab for a period of time mid-treatment, but hemoglobin concentrations increased after both the first and second courses of adalimumab. Continuous and uninterrupted treatment with adalimumab may be more efficacious, as has been found in both psoriasis and Crohn's disease [19,20].

## Conclusion

This is the first report describing the use of adalimumab in a patient with systemic sarcoidosis with bone marrow involvement. TNF antagonism with adalimumab was efficacious and well-tolerated in this patient and may be considered as a treatment option for similar cases. Notably, this patient's anemia improved both with initial treatment and after re-initiation of adalimumab therapy. The results of this case also demonstrate that adalimumab may be steroid sparing, as our patient was able to stop prednisone therapy completely. Conclusions drawn from a single case report must be interpreted with caution and considered tentative until additional cases and randomized controlled trials are performed to evaluate efficacy, safety, and appropriate dosing of adalimumab in patients with sarcoidosis.

## Abbreviations

CT: computed tomography; PPD: purified protein derivative; TNF: tumor necrosis factor.

## Consent

Written informed consent was obtained from the patient for publication of this case report and any accompanying images. A copy of the written consent is available for review by the Editor-in-Chief of this journal.

## Competing interests

The author declares that he has no competing interests.
